# Silicone Rubber Composites with High Breakdown Strength and Low Dielectric Loss Based on Polydopamine Coated Mica

**DOI:** 10.3390/polym11122030

**Published:** 2019-12-07

**Authors:** Yifan Liao, Yunxuan Weng, Jiaqi Wang, Hongfu Zhou, Jun Lin, Shaojian He

**Affiliations:** 1 School of Electric Power, South China University of Technology, Guangzhou 510640, China; 2 Electric Power Research Insititute, China Southern Power Grid Co., Ltd., Guangzhou 510080, China; 3 Beijing Key Laboratory of Quality Evaluation Technology for Hygiene and Safety of Plastics, Beijing 100048, China; 4 School of Renewable Energy, North China Electric Power University, Beijing 102206, China

**Keywords:** silicone rubber, mica, polydopamine, vinyl tri-methoxysilane, dielectric loss, breakdown strength

## Abstract

High breakdown strength and low dielectric loss are necessary for the outdoor insulator using silicone rubber (SR) composites. In this work, polydopamine coated mica (mica-PDA) was synthesized via bioinspired dopamine self-polymerization, and mica-PDA-filled SR composite (SR/mica-PDA-VTMS) was prepared using vinyl tri-methoxysilane (VTMS) as a silane coupling agent which serves as the molecular bridges between the organic rubber and the inorganic filler. The SR/mica-PDA-VTMS composite demonstrated dense and uniform morphology where the filler was well dispersed. Due to the strong interfacial interactions between filler and rubber, the SR/mica-PDA-VTMS composite exhibits much lower dielectric loss compared to the other mica-filled SR composites, which was comparable to the prepared alumina-tri-hydrate-filled SR composites. Moreover, the breakdown strength of ~31.7 kV/mm and tensile strength of 5.4 MPa were achieved for the SR/mica-PDA-VTMS composite, much higher than those of the other as-prepared SR composites.

## 1. Introduction

Silicone rubber (SR) has been widely used in high-voltage outdoor insulation due to the excellent hydrophobicity and weather-resistance [1,2,3]. Fillers are added into the pure SR to improve specific properties as well as to reduce cost [4,5,6]. So far, alumina tri-hydrate (ATH) is the most commonly used filler in the SR composite insulator [7,8]. However, the release of the hydrate from ATH can roughen the surface of the composite insulator causing further wetting and dry band arcing under long-term operation [9,10]. Therefore, it is of great importance to find substitute fillers to replace ATH applied in the SR composite insulator. As for SR composites applied in high-voltage insulation, the breakdown strength and dielectric loss are the two most important properties.

Mica is a natural mineral composed of a layered silicate structure with excellent electrical insulating properties, which has played a role as the reinforcement filler in different types of polymer composites [11,12,13]. However, due to the hydrophilicity, when mica is used as filler in the SR matrix, the poor filler-rubber compatibility could lead to the high dielectric loss, which limits the application of mica in the electrical insulation field. Silane coupling agents are the most popular surface modifier for the inorganic filler to improve the compatibility between the hydrophilic filler and hydrophobic rubber, further, to improve the performance of rubber composites [14,15,16]. Unfortunately, the active sites on the mica surface that could react with silane coupling agents are relatively few. Dopamine derivatives were reported to self-polymerize to form polydopamine, which can adhere to different kinds of materials [17,18,19,20], thus introducing plenty of catechol group active sites. Previously, we have successfully coated some inorganic fillers with polydopamine, which were further modified with secondary reaction because of the existing catechol groups of polydopamine [21,22]. Some recent reports also confirmed that polydopamine could be easily coated onto the surface of the nanoparticles [23,24,25].

In this work, mica was first coated with polydopamine to obtain mica-PDA, rendering mica with a large number of hydroxyl groups which react more easily with the silane coupling agent. Mica-PDA-filled SR composites (SR/mica-PDA-VTMS) were prepared with mica-PDA as a filler and with vinyl tri-methoxysilane (VTMS) as a silane coupling agent. For comparison, some other SR composites were also prepared and investigated to reveal the structure–property relationship of these SR composites.

## 2. Experimental

### 2.1. Materials

SR (MVQ110, vinyl content: 0.23 wt%, molecular weight obtained from viscosity measurement: 680,000 g/mol) was provided by Zhejiang Xin’an Chemical Group Co. Ltd., Xin’an, China. Mica (97%) was provided by Jinghang Mineral Products Co. Ltd., Lingshou, China. ATH (97%) was obtained from Shandong Seibou Chemical Technology Co. Ltd., Zibo, China. VTMS (99%) was purchased from Shuguang Chemical Co. Ltd., Nanjing, China. Dopamine hydrochloride (99%), sodium hydroxide (98%), and tris(hydroxymethyl)-aminomethane hydrochloride (Tris-HCl, 99%) were purchased from Alfa Aesar, Tianjin, China. 2,5-bis(tert-butylperoxy)-2,5-dimethyl hexane (DBPMH, 97%) was obtained from Akzo Nobel Cross-Linking Peroxide Co. Ltd., Suzhou, China. All the materials were used as received.

### 2.2. Preparation

Preparation of PDA-mica: 40 g mica was dispersed in 1 L water ultrasonically for 2 h, and then 0.375 g dopamine hydrochloride and 0.045 g Tris-HCl were added into the mixture, followed by adjusting pH to 8.5 using sodium hydroxide. The suspension mixture was stirred at room temperature for 12 h followed by being centrifuged and rinsed several times with water to obtain mica-PDA. Mica-PDA was dried in vacuum at 50 °C overnight before use.

Preparation of SR composites: filler (including ATH, mica, or mica-PDA) and other ingredients were mixed into the gum SR on a 6-in. two-roll mill to prepare SR compounds. To obtain SR composites, the compounds were cured in a standard mold using a hydraulic hot press at 160 °C and 15 MPa. The recipe (parts by weight) for the sample preparation was as follows: SR, 100.0; 2,5-bis(tert-butylperoxy)-2,5-dimethyl hexane (DBPMH), 0.5; filler, 150; VTMS, 0 or 1.5).

### 2.3. Characterization

X-ray photoelectron spectroscopy (XPS) (ESCALAB 250, Thermo Fisher Scientific, Rochester, NY, USA) with a 150 W monochromatic Al Kα radiation was used to detect the chemical composition of the samples. The morphologies of the fillers and composites were observed by scanning electron microscopy (SEM) (SU8010, Hitachi Co. Ltd., Tokyo, Japan) at an accelerating voltage of 5 kV. The dielectric properties were measured using an impedance analyzer (6500B, Wayne Kerr Electronics, Shenzhen, China). Breakdown strength was recorded by a dielectric strength tester (HCDJC-50kV, Beijing Huace Testing Instrument Co. Ltd., Beijing, China) with a stepping voltage of 1 kV/s according to IEC 60243-1-2013, and equal-diameter electrodes with a diameter of 25 mm and samples with a thickness of 1 mm were used. Stress–strain test was performed on an electrical tensile tester (AI-7000S1, Goodtechwill Testing Machines, Co. Ltd., Qingdao, China) at a speed of 500 mm·min^−1^. All the measurements were conducted at 25 °C.

## 3. Results and Discussion

### 3.1. Structure Characterization

**Figure 1:**
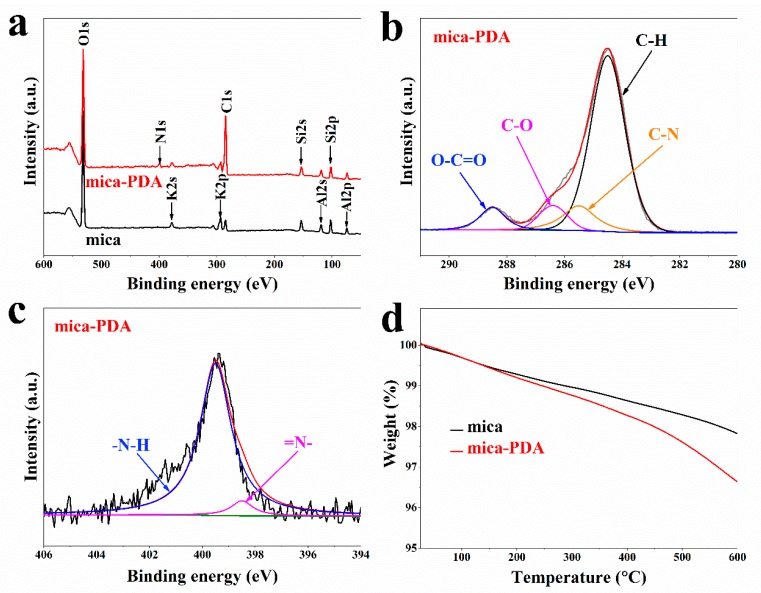
(**a**) X-ray photoelectron spectroscopy (XPS) survey scan spectra of mica and polydopamine coated mica (mica-PDA), (**b**) C1s, and (**c**) N1s core-level spectra of mica-PDA; (**d**) TGA curves of mica and mica-PDA.

To effectively graft VTMS on the filler, polydopamine was coated on the surface of mica to provide more active sites (catechol groups). The chemical composition of mica-PDA XPS was probed by XPS. As shown in Figure 1a, as compared to mica, mica-PDA exhibits the characteristic N1s peak, indicating the deposition of the polydopamine coating on the filler, which is also confirmed by C1s and N1s core-level spectra. For the curved fitted C1s core-level spectrum of mica-PDA shown in Figure 1b, there are four peak components with the binding energy at 284.6 eV, 285.5 eV, 286.4 eV, and 288.5 eV, representing the C–H species, C–N species, C-O species, and O-C=O species, respectively [26,27,28]. Meanwhile, for the N1s core-level spectrum of mica-PDA shown in Figure 1c, two peak components at 398.5 eV and 399.5 eV are attributed to –N= species and –N–H species, respectively, which are formed by the indole groups via structure evolution during dopamine self-polymerization [29,30]. Furthermore, as shown in the TGA curves of mica and mica-PDA (Figure 1d), both mica and mica-PDA show the similar degradation behavior, while the weight loss between 200 and 600 °C for mica-PDA is slightly larger than that of mica due to the additional degradation from polydopamine. At 600 °C, mica and mica-PDA show a weight loss of 2.0% and 3.5%, respectively, indicating that the weight content of polydopamine in mica-PDA is 1.5%. Therefore, both XPS and TGA results confirm that polydopamine was successfully coated on the surface of mica in mica-PDA.

**Scheme 1:**
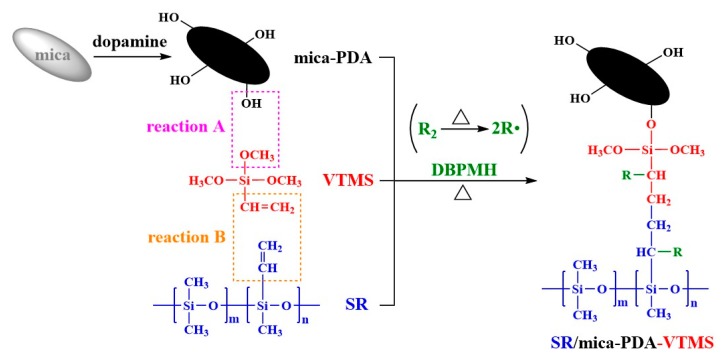
Mechanism of the formation of mica-PDA-filled silicone rubber (SR) composite (SR/mica-PDA-VTMS).

To prepare SR/mica-PDA-VTMS composites, mica-PDA (filler), VTMS (silane coupling agent), and DBPMH (curing agent) were added into SR through mechanical blending, followed by being cured at high temperature. Since catechol group active sites (through self-polymerized dopamine) have been introduced on mica-PDA, the reaction between the catechol groups and VTMS occurs (Sketch 1. reaction A), which results in the immobilization of the silane coupling agent on mica-PDA. At high temperature, the decomposition of DBPMH produces free radicals, which initiate the radical addition reactions between the –CH=CH_2_ groups on both SR and VTMS (Scheme 1. reaction B). As a result, mica-PDA filler particles should be chemically combined with the SR macromolecular chains, strengthening the filler–rubber interactions, as shown in Sketch 1.

**Figure 2:**
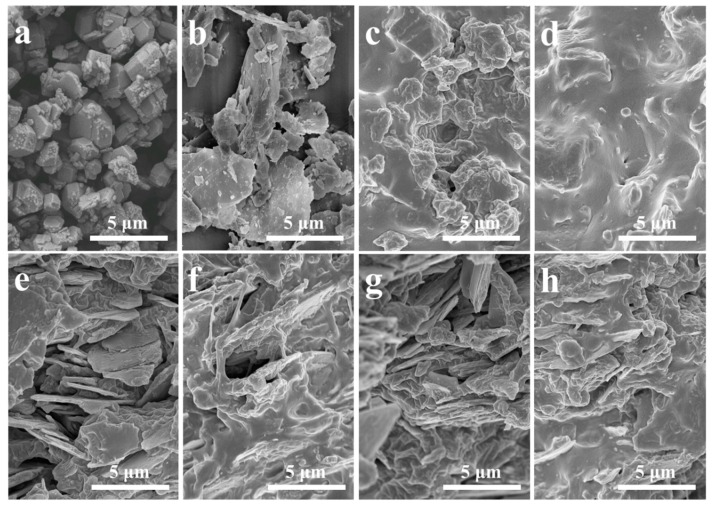
SEM images of (**a**) alumina tri-hydrate (ATH), (**b**) mica, and the fractured surface morphologies for (**c**) SR/ATH, (**d**) SR/ATH-vinyl tri-methoxysilane (VTMS), (**e**) SR/mica, (**f**) SR/mica-VTMS, (**g**) SR/mica-PDA, and (**h**) SR/mica-PDA-VTMS composites.

SEM images of ATH and mica powder are shown in Figure 2a,b, it can be seen that the ATH particles with a diameter of ~2 μm possess the relative regular structure as compared to the mica plates with a length of ~5 μm. It is easy to distinguish ATH and mica from the shape of the particles even when either of them is embedded in the SR matrix. SEM images of the tensile fracture surfaces for the prepared SR composites are shown in Figure 2c–h. It is found that the ATH particles’ aggregates are exposed on the fracture surfaces and some voids are observed in the SR/ATH composite (Figure 2c), indicating the weak adhesive force between SR and ATH. For comparison, SR/ATH-VTMS composite (Figure 2d) displays fewer aggregates and voids resulting from the improved interfacial interactions between filler and rubber with the assistance of VTMS. A similar comparison is also found between the SR/mica (Figure 2e) and SR/mica-VTMS composites (Figure 2f), confirming the enhanced interfacial interactions between filler and rubber by adding VTMS. Nevertheless, there exist more voids and larger cracks between the interfaces for the SR/mica-VTMS composite compared to those for the SR/ATH-VTMS composite. This could be attributed to the following factors: (i) the aspect ratio of mica is larger than that of ATH, which makes it more difficult to be covered by rubber macromolecules; (ii) compared with ATH, mica has fewer active sites on the surface that can react with VTMS, resulting in the poorer silane coupling modification effect. After being coated by polydopamine on the surface of mica, filler dispersion is improved for the SR/mica-PDA composite (Figure 2g) because of the improved compatibility between the filler and rubber [31]. However, due to the physical interface, the interactions between filler and rubber are still too weak. Meanwhile, for the SR/mica-PDA-VTMS composite (Figure 2h), a significant improvement on both filler dispersion and interfacial interactions are confirmed through morphology observation. Such improvement should be attributed from the formation of the chemical binding between filler and rubber, which has been illustrated in Sketch 1.

### 3.2. Performance of SR Composites

**Figure 3:**
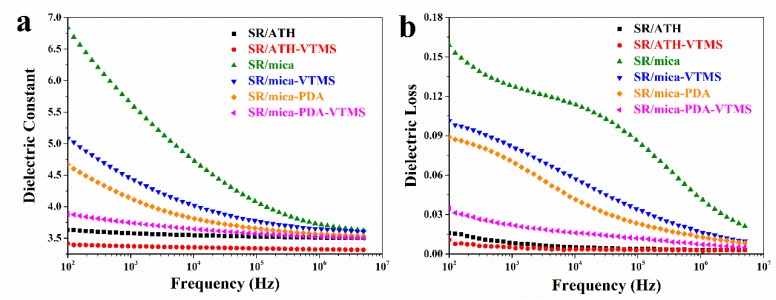
(**a**) Dielectric constant and (**b**) dielectric loss of SR composites.

The dielectric constant and dielectric loss of the SR composites are presented in Figure 3a,b, respectively. Since ATH shows much smaller aspect ratio than mica, the ATH-filled SR composites exhibit the lower dielectric constant and dielectric loss than all the mica-filled SR composites. Both dielectric constant and dielectric loss decrease by incorporating VTMS in either SR/ATH or SR/mica composites. For the four different mica-filled SR composites, both dielectric constant and dielectric loss follows the order of SR/mica-PDA-VTMS < SR/mica-PDA < SR/mica-VTMS < SR/mica, with SR/mica-PDA-VTMS composite showing much lower dielectric loss than the other composites. Such behavior is related to the filler–rubber interactions in the composites, which significantly affect interfacial polarization. Generally, the strong filler–rubber interactions could reduce the interfacial polarization, resulting in the lower dielectric constant and dielectric loss. In particular, SR/mica-PDA composite with physical interfacial interactions shows a slightly lower dielectric constant and dielectric loss than SR/mica-VTMS composite. Such result could be ascribed to the better filler dispersion in the SR/mica-PDA composite, as demonstrated in the abovementioned morphologies observation. Using this promising modification method, the SR/mica-PDA-VTMS composite with low dielectric loss can be obtained, which is comparable to the ATH-filled SR composites.

**Figure 4:**
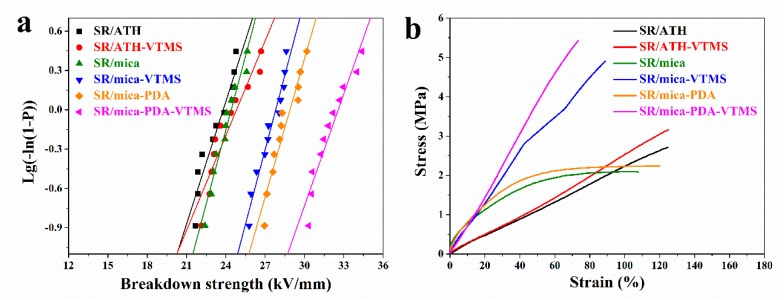
(**a**) Weibull distribution of breakdown strength and (**b**) stress–strain curves for SR composites.

For the mica-filled SR composites, when being exposed under a high-voltage electric field, the large-aspect-ratio mica plates could act as barriers inhibiting the propagation of electrical treeing [32], which is beneficial to breakdown strength. However, there exist interfaces between the filler phase and the rubber phase, more compatibility between the two phases would result in fewer defects in the interfaces. The insulation material with more defects would result in more space-charges accumulation inside [33], and thus decrease the breakdown strength. For mica-filled SR composite, there exist more defects inside compared to ATH-filled SR composite, as illustrated in Figure 2, which is harmful to the breakdown strength. The interfacial modification could decrease the defects in the composites, thus improving the breakdown strength. Therefore, as shown in the Weibull distribution of breakdown strength in Figure 4a, the breakdown strength of the mica-filled SR composites follows the order of SR/mica-PDA-VTMS > SR/mica-PDA > SR/mica-VTMS > SR/mica, which is closely related to defects observed in the morphologies of the composites, as shown in Figure 2e–h. Meanwhile, for the ATH-filled SR composites, there is only little increase in the breakdown strength when incorporating VTMS due to the fewer defects in the SR/ATH composite than in the SR/mica composite. The best breakdown strength of ~31.7 kV/mm is achieved for SR/mica-PDA-VTMS composite, much higher than that of SR/ATH-VTMS composite (~22.1 kV/mm), where the main components have been widely used in the commercial silicone rubber insulator.

Moreover, the mechanical performance of the SR composites are compared in Table 1 and the corresponding stress–strain curves are presented in Figure 4b. A slight increase in tensile strength of the ATH-filled SR composites is found by adding VTMS (from 2.7 MPa to 3.2 MPa), while the enhancement of tensile strength for mica-filled SR composite is more remarkable (from 2.1 MPa to 4.9 MPa), which might be due to the different filler shapes. As for the SR/mica-PDA composite, it shows a little bit better performance in tensile strength and elongation at break than the SR/mica composite due to the improvement of filler–rubber compatibility but still physical interactions. The best tensile strength of 5.4 MPa is achieved for the SR/mica-PDA-VTMS composite, which should be ascribed from the better filler dispersion and stronger interfacial interactions.

## 4. Conclusions

In this work, mica was coated with polydopamine to introduce a large number of hydroxyl groups that react more easily with the silane coupling agent. As illustrated in the SEM observation, the stronger filler–rubber interactions could result in better filler dispersion and fewer voids in the composites. Four different mica-filled SR composites and two different ATH-filled SR composites were prepared and investigated. SR/mica-PDA-VTMS composite showed much lower dielectric loss than the other mica-filled SR composites, comparable to those of the ATH-filled SR composites. The breakdown strength of the four mica-filled SR composites was closely related to the filler–rubber interactions as well as the defects observed in the morphologies of the composites. Owing to the good filler dispersion and strong filler–rubber interactions, the best performance was achieved for the SR/mica-PDA-VTMS, with breakdown strength of ~31.7 kV/mm and tensile strength of 5.4 MPa, much higher than those of the ATH-filled SR composites which have been widely used in the commercial composite insulator. Our work indicated that mica would be a promising filler applied in the field of high-voltage insulation.

## Figures and Tables

**Table 1:** Mechanical properties of SR composites.

Samples	Elastic Modulus (MPa)	Tensile Strength (MPa)	Elongation at Break (%)
SR/ATH	0.14	2.7	124
SR/ATH-VTMS	0.16	3.2	125
SR/mica	0.54	2.1	108
SR/mica-VTMS	0.35	4.9	89
SR/mica-PDA	0.56	2.2	120
SR/mica-PDA-VTMS	0.43	5.4	73

## References

[1] Plesa I., Notingher P.V., Schloegl S., Sumereder C., Muhr M. (2016). Properties of polymer composites used in high-voltage applications. Polymers.

[2] Li S., Yu S., Feng Y. (2016). Progress in and prospects for electrical insulating materials. High Volt..

[3] Wu C., Gao Y., Liang X., Gubanski S.M., Wang Q., Bao W., Li S. (2019). Manifestation of Interactions of Nano-Silica in Silicone Rubber Investigated by Low-Frequency Dielectric Spectroscopy and Mechanical Tests. Polymers.

[4] Xue Y., Li X.F., Wang H.S., Zhang D.H., Chen Y.F. (2019). Thermal conductivity improvement in electrically insulating silicone rubber composites by the construction of hybrid three-dimensional filler networks with boron nitride and carbon nanotubes. J. Appl. Polym. Sci..

[5] Nazir M.T., Phung B.T., Yu S., Zhang Y., Li S. (2018). Tracking, erosion and thermal distribution of micro-AlN + nano-SiO_2_ co-filled silicone rubber for high-voltage outdoor insulation. High Volt..

[6] Han T., Du B., Su J., Gao Y., Xing Y., Fang S., Li C., Lei Z. (2019). Inhibition Effect of Graphene Nanoplatelets on Electrical Degradation in Silicone Rubber. Polymers.

[7] Ansorge S., Schmuck F., Papailiou K.O. (2015). Impact of different Fillers and Filler Treatments on the Erosion Suppression Mechanism of Silicone Rubber for Use as Outdoor Insulation Material. IEEE Trans. Dielectr. Electr. Insul..

[8] Momen G., Farzaneh M. (2011). Survey of micro/nano filler use to improve silicone rubber for outdoor insulators. Rev. Adv. Mater. Sci..

[9] Kim S.H., Cherney E.A., Hackam R. (1992). Effects of filler level in RTV silicone rubber coatings used in HV insulators. IEEE Trans. Electr. Insul..

[10] Meyer L.H., Cherney E.A., Jayaram S.H. (2004). The role of inorganic fillers in silicone rubber for outdoor insulation alumina tri-hydrate or silica. IEEE Electr. Insul. Mag..

[11] Tanaka K., Kojima H., Onoda M., Suzuki K. (2015). Prediction of Residual Breakdown Electrical Field Strength of Epoxy-Mica Paper Insulation Systems for the Stator Winding of Large Generators. IEEE Trans. Dielectr. Electr. Insul..

[12] Cui X., Zhu G., Liu W. (2017). Synthesis, characterisation and enhanced properties of polyimide/mica hybrid films. Plast. Rubber Compos..

[13] Wang J., Xie Y.C., Liu J.J., Zhang Z.C., Zhang Y.F. (2019). Towards high efficient nanodielectrics from linear ferroelectric P(VDF-TrFE-CTFE)-g-PMMA matrix and exfoliated mica nanosheets. Appl. Surf. Sci..

[14] He S.J., Hu J.B., Zhang C., Wang J.Q., Chen L., Bian X.M., Lin J., Du X.Z. (2018). Performance improvement in nano-alumina filled silicone rubber composites by using vinyl tri-methoxysilane. Polym. Test..

[15] He S.J., Wang J.Q., Hu J.B., Zhou H.F., Nguyen H., Luo C.M., Lin J. (2019). Silicone rubber composites incorporating graphitic carbon nitride and modified by vinyl tri-methoxysilane. Polym. Test..

[16] He S.J., Xue Y., Lin J., Zhang L.Q., Du X.Z., Chen L. (2016). Effect of silane coupling agent on the structure and mechanical properties of nano-dispersed clay filled styrene butadiene rubber. Polym. Compos..

[17] Wan X., Zhan Y., Long Z., Zeng G., Ren Y., He Y. (2017). High-performance magnetic poly (arylene ether nitrile) nanocomposites: Co-modification of Fe_3_O_4_ via mussel inspired poly(dopamine) and amino functionalized silane KH550. Appl. Surf. Sci..

[18] Yang D., Ruan M.N., Huang S., Wu Y.B., Li S.X., Wang H., Shang Y.W., Li B.Y., Guo W.L., Zhang L.Q. (2016). Improved electromechanical properties of NBR dielectric composites by poly (dopamine) and silane surface functionalized TiO_2_ nanoparticles. J. Mater. Chem. C.

[19] Liu Y., Ai K., Lu L. (2014). Polydopamine and Its Derivative Materials: Synthesis and Promising Applications in Energy, Environmental, and Biomedical Fields. Chem. Rev..

[20] Ryu J.H., Messersmith P.B., Lee H. (2018). Polydopamine Surface Chemistry: A Decade of Discovery. ACS Appl. Mater. Interfaces.

[21] Liu X., He S.J., Song G., Jia H.N., Shi Z.Z., Liu S.X., Zhang L.Q., Lin J. (2016). Proton conductivity improvement of sulfonated poly (ether ether ketone) nanocomposite membranes with sulfonated halloysite nanotubes prepared via dopamine-initiated atom transfer radical polymerization. J. Membr. Sci..

[22] He S.J., Liu S.X., Dai W.X., Zhai S.X., Lin J. (2018). Nanocomposite proton exchange membranes incorporating phosphotungstic acid anchored on imidazole-functionalized halloysite nanotubes. J. Electrochem. Soc..

[23] Zheng Y., Wang X., Wu G. (2019). Facile Strategy of Improving Interfacial Strength of Silicone Resin Composites Through Self-Polymerized Polydopamine Followed via the Sol-Gel Growing of Silica Nanoparticles onto Carbon Fiber. Polymers.

[24] Massaro M., Armetta F., Cavallaro G., Martino D.F.C., Gruttadauria M., Lazzara G., Riela S., d’ischia M. (2019). Effect of halloysite nanotubes filler on polydopamine properties. J. Colloid Interface Sci..

[25] Jiang W., Zhang X., Luan Y., Wang R., Liu H., Li D., Hu L. (2019). Using γ-Ray Polymerization-Induced Assemblies to Synthesize Polydopamine Nanocapsules. Polymers.

[26] Zhu L., Lu Y., Wang Y., Zhang L., Wang W. (2012). Preparation and characterization of dopamine-decorated hydrophilic carbon black. Appl. Surf. Sci..

[27] Yang D., Huang S., Ruan M., Li S., Yang J., Wu Y., Guo W., Zhang L. (2018). Mussel Inspired Modification for Aluminum Oxide/Silicone Elastomer Composites with Largely Improved Thermal Conductivity and Low Dielectric Constant. Ind. Eng. Chem. Res..

[28] Ruan M., Yang D., Guo W., Zhang L., Wu Y., Li S. (2018). Mussel-inspired synthesis of barium titanate @ poly (dopamine) @ graphene oxide multilayer core-shell hybrids for high-performance dielectric elastomer actuator. Mater. Lett..

[29] He S.J., Dai W.X., Yang W., Liu S.X., Bian X.M., Zhang C., Lin J. (2019). Nanocomposite proton exchange membranes based on phosphotungstic acid immobilized by polydopamine-coated halloysite nanotubes. Polym. Test..

[30] Ruan M., Yang D., Guo W., Zhang L., Li S., Shang Y., Wu Y., Zhang M., Wang H. (2018). Improved dielectric properties, mechanical properties, and thermal conductivity properties of polymer composites via controlling interfacial compatibility with bio-inspired method. Appl. Surf. Sci..

[31] He S.J., He T.F., Wang J.Q., Wu X.H., Xue Y., Zhang L.Q., Lin J. (2019). A novel method to prepare acrylonitrile-butadiene rubber/clay nanocomposites by compounding with clay gel. Compos. Part B Eng..

[32] Li X.F., Zhang D.H., Chen Y.F. (2017). Silicone rubber/hollow silica spheres composites with enhanced mechanical and electrical insulating performances. Mater. Lett..

[33] Xue Y., Li X.F., Zhang D.H., Wang H.S., Chen Y., Chen Y.F. (2018). Comparison of ATH and SiO_2_ fillers filled silicone rubber composites for HTV insulators. Compos. Sci. Technol..

